# Transcriptional Regulation Buffers Gene Dosage Effects on a Highly Expressed Operon in *Salmonella*

**DOI:** 10.1128/mBio.01446-18

**Published:** 2018-09-11

**Authors:** Eva Garmendia, Gerrit Brandis, Diarmaid Hughes

**Affiliations:** a Department of Medical Biochemistry and Microbiology, Uppsala University, Uppsala, Sweden; Emory University

**Keywords:** chromosome organization, EF-Tu, location bias, *tufB*

## Abstract

Highly expressed genes are commonly located close to the origin of replication of bacterial chromosomes (OriC). This location skew is thought to reflect selective advantages associated with gene dosage effects during the replication cycle. The expression of constitutively expressed genes can vary up to fivefold based on chromosomal location, but it is not clear what level of variation would occur in naturally regulated operons. We tested the magnitude of the chromosome location effect using EF-Tu (*tufA*, *tufB*), an abundant protein whose cellular level correlates with, and limits, the maximum growth rate. We translocated the *Salmonella tufB* operon to four locations across the chromosome. The distance from OriC had only a small effect on growth rate, consistent with this operon having the natural ability to upregulate expression and compensate for reduced gene dosage. In contrast, when the total EF-Tu concentration was limiting for the growth rate (*tufA* deleted), we observed a strong gene dosage effect when *tufB* was located further from OriC. However, only a short period of experimental evolution was required before the bacteria adapted to this EF-Tu starvation situation by acquiring genetic changes that increased expression levels from the translocated *tufB* gene, restoring growth rates. Our findings demonstrate that, at least for the *tufB* operon, gene dosage is probably not the dominant force selecting for a chromosomal location close to OriC. We suggest that the colocation of highly expressed genes close to OriC might instead be selected because it enhances their coregulation during various growth states, with gene dosage being a secondary benefit.

## Abstract (precis)

A feature of bacterial chromosomes is that highly expressed essential genes are usually located close to the origin of replication. Because bacteria have overlapping cycles of replication, genes located close to the origin will often be present in multiple copies, and this is thought to be of selective benefit where high levels of expression support high growth rate. However, the magnitude of this selective effect and whether other forces could be at play are poorly understood. To study this, we translocated a highly expressed essential operon, *tufB*, to different locations and measured growth fitness. We found that transcriptional regulation buffered the effects of translocation and that even under conditions where growth rate was reduced, genetic changes that increased the expression of *tufB* were easily and rapidly selected. We conclude, at least for *tufB*, that forces other than gene dosage may be significant in selecting for chromosomal location.

## INTRODUCTION

A common feature of bacterial genomes is that the organization of genes on the chromosome is often highly skewed with respect to location relative to the origin of replication. In free-living bacteria, highly expressed genes involved in transcription and translation are generally located close to the origin of replication (1, 2). This locational bias is thought to reflect a selection for maximum growth rate that can be achieved by increasing relative gene copy number and, therefore, expression, due to gene dosage effects associated with bacteria having overlapping cycles of chromosome replication under fast growth conditions (3–6). While genome sequence analyses provide overwhelming evidence that gene location bias is an important feature of bacterial genomes, they leave unanswered questions concerning the magnitude and nature of the selective forces underlying this situation. Several studies have quantified the gene dosage effect experimentally and shown that the levels of expression of genes vary up to fivefold depending on their chromosomal location (5, 7, 8). Changes of that order of magnitude in expression of important proteins, such as ribosomal proteins or elongation factors, would lead to a significant reduction in bacterial growth rate (9, 10), potentially explaining the strong selection for gene location on the chromosome. While demonstrating the effect of gene copy number on expression level, a major caveat in previous studies has been that they focused only on expression levels of constitutively expressed genes. These studies have, for example, used a mutated version of the *his* operon (5) or an isopropyl-β-d-thiogalactopyranoside (IPTG)-induced *lac* promoter (8) that under natural circumstances are feedback regulated (11, 12). Thus, it is not clear if the demonstrated effects would be observed for these genes under natural circumstances. Furthermore, the genes assayed for expression in these previous studies were not genes that are under selection for determining maximum bacterial growth rate. Accordingly, we currently have very limited experimental evidence supporting selection for maximum growth rates by increasing relative gene copy number as the primary driver for gene location, and we have little or no quantitative understanding of the magnitude of the selective forces involved.

To experimentally address and quantify the effect of gene location on bacterial growth rate, we decided to move a highly expressed and highly conserved operon to different locations within a bacterial chromosome. The chosen operon, the *tufB* operon of *Salmonella* Typhimurium (proper name, Salmonella enterica serovar Typhimurium LT2), has its native location approximately 276 kb from the origin of replication (the total distance separating the origin from the terminus is approximately 2,400 kb), and its expression is directly linked with determining the maximum bacterial growth rate (9). The *tufB* operon encodes four tRNA genes (*thrU*, *tyrU*, *glyT*, and *thrT*) and *tufB*, encoding the translation elongation factor EF-Tu. Two of the tRNA genes, *thrU* and *glyT*, are probably essential for growth, being the only tRNAs in the genome predicted to read ACA and GGA codons, respectively (13). In *Salmonella*, there are two separated genes, *tufA* and *tufB*, that encode identical EF-Tu proteins (14). They are located on opposite sides of the origin of replication, approximately equidistant from the origin, each oriented for transcription in the same direction as replication. Under fast growth conditions, EF-Tu in *Salmonella* accounts for 9% of total cytoplasmic protein (9), similar to Escherichia coli where the genetic organization is identical (15, 16) and where EF-Tu has been quantified as the most abundant cytoplasmic protein under fast growth conditions (17). EF-Tu functions in protein synthesis by forming ternary complexes with each elongator tRNA and bringing them into contact with the ribosomal A-site to drive fast and accurate protein synthesis (18–20). Reductions in EF-Tu concentration or in EF-Tu activity are closely correlated with reductions in bacterial growth rate (9). The huge demand for high concentrations of EF-Tu to drive fast protein synthesis is consistent with the genetic locations of the *tuf* genes under the gene dosage hypothesis. These functional and genetic features make the *tufB* operon a good candidate for testing the phenotypic consequences of altering gene location.

Here, we moved the *tufB* operon of *Salmonella* to four nonnative chromosomal locations across the bacterial chromosome and showed that the displacement of this highly expressed operon had a smaller effect on bacterial growth rates than expected by the gene dosage hypothesis. We were able to show that strong gene dosage effects were visible under EF-Tu starvation conditions (when *tufA* was additionally removed from the chromosome), but a short-term evolution experiment showed that this effect could be ameliorated by genetic changes that increased expression from the *tufB* operon. We conclude that gene dosage can be a strong selective force on the naturally regulated *tufB* operon when growth rate is extremely limited by its expression level (when its expression is constitutively at its maximum). Since this is rarely the case under natural conditions and genetic changes to increase expression can easily ameliorate the effect, we suggest that other forces, for example, coregulation of highly expressed genes during various growth states, might play a more important role in the selection of gene location than high expression level *per se*.

## RESULTS

### The *tufB* operon has the ability to buffer gene dosage effects.

**FIG 1:**
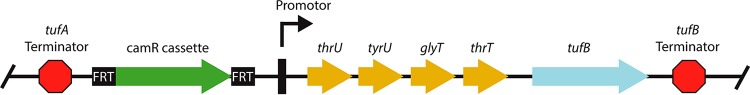
Schematic representation of the custom-made *tufB* operon. This operon was inserted by recombineering with selection at different chromosomal locations.

To place the *tufB* operon (promoter region followed by four tRNA genes, followed by *tufB*) at different locations on the chromosome of *S. Typhimurium*, we constructed a custom-made *tufB* operon with a terminator sequence from *tufA* followed by an FRT-*cat*-FRT (where FRT stands for FLP recombination target) cassette upstream of the regulatory region of the native *tufB* operon (Fig. 1). This construct is designed to insulate the *tufB* operon from any transcriptional activity in the surrounding chromosomal sequences and to be selectable by its chloramphenicol-resistant phenotype.

**FIG 2:**
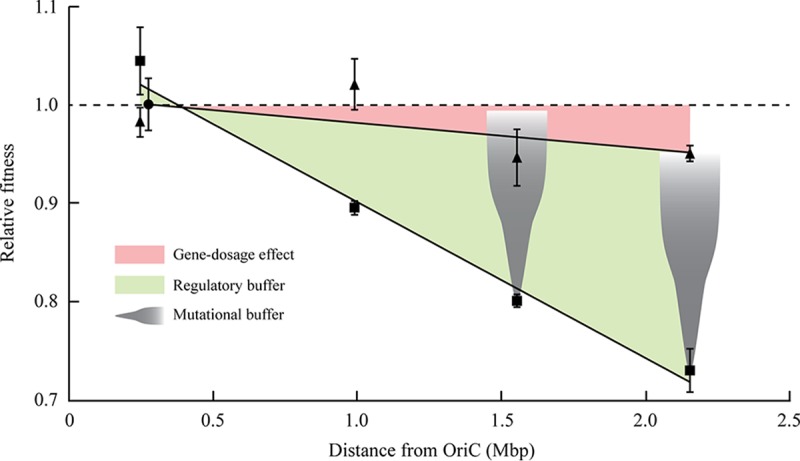
Relative fitness as a function of chromosomal location of the *tufB* operon in strains with a *tufA* gene present (triangle) or deleted (square). All values are relative to the respective isogenic strain with the *tufB* operon at its native location (circle). The red area indicates the effect of gene dosage on bacterial fitness, and the green area indicates the regulatory buffer of the *tufB* gene by which upregulation of gene expression decreases the effect of reduced gene dosage. The gray cones show the mutational buffer by which genetic alteration can upregulate gene expression and, thereby, decrease the effect of reduced gene dosage.

To assess whether the location of the *tufB* operon affected the bacterial fitness, we placed the custom-made *tufB* operon at four different genomic locations, all except one at a greater distance from the origin of replication than the native *tufB* operon (Table 1). After successful translocation of the *tufB* operon into each of the nonnative locations, we removed the *cat* gene from the resistance cassette by expressing the *flp* protein and deleted the entire native *tufB* operon. The native *tufB* operon terminator was left in place due to its regulatory role important for expression of the downstream operon. The resulting set of five isogenic strains (*tufB* operon located in the native location and four nonnative locations) was confirmed by whole-genome sequencing, and exponential growth rates were measured to determine the relative fitness of the constructed strains. Based on distance from the origin of replication, the relative gene dosage of *tufB* in bacteria growing in rich medium was expected to vary from 1 (native location) down to approximately 0.2 (most distant location). Interestingly, translocation of the *tufB* operon had less of an effect on bacterial fitness than predicted by changes in gene dosage, with no fitness cost for the two locations closest to the native *tufB* operon and a fitness cost of 5% for the two locations closest to the terminus of replication (Table 1 and Fig. 2). We measured *tufB* expression levels based on a transcriptional *tufB*-*yfp* fusion assay (Materials and Methods) for each of the five chromosomal locations to test whether operon translocation affects *tufB* expression levels. The results show no significant change in *tufB* expression levels for the two locations closest to the native *tufB* operon and, in agreement with the fitness data, a 7 to 9% reduction in *tufB* expression for the two locations closest to the terminus of replication (Table 1).

**TABLE 1:** Relative fitness of strains carrying the *tufB* operon at different locations and orientations

Location^*a*^	Relative fitness^*b*^		Relative *tufB* expression^*c*^	
	wt	Δ*tufA*	wt	Δ*tufA*
246	0.98 ± 0.02	1.04 ± 0.03	1.06 ± 0.04	1.28 ± 0.02
276^*d*^	1.00 ± 0.03	1.00 ± 0.02	1.00 ± 0.04	1.00 ± 0.01
991	1.02 ± 0.03	0.90 ± 0.01	0.99 ± 0.03	0.91 ± 0.01
1555	0.95 ± 0.03	0.80 ± 0.01	0.93 ± 0.01	0.82 ± 0.02
2154	0.95 ± 0.01	0.73 ± 0.02	0.91 ± 0.03	0.71 ± 0.01

a Chromosomal location as the distance to OriC in kilobases.

b Relative fitness ± standard deviation measured as the exponential growth rate. wt, wild type.

c Relative *tufB* expression level ± standard deviation measured as transcriptional *yfp* fusion in a wild-type (wt) or Δ*tufA* strain. Values are relative to the values for their respective isogenic strains with the *tufB* operon in the original location.

d Native *tufB* operon location.

### Gene dosage effects are visible under extreme EF-Tu starvation conditions.

**FIG 3:**
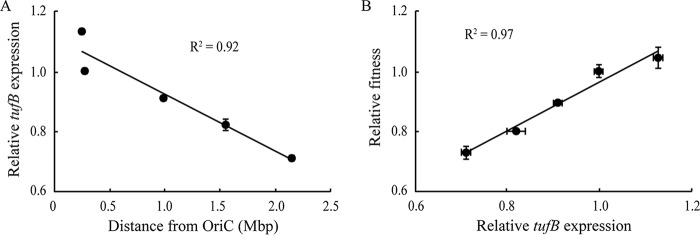
(A and B) Relative *tufB* expression as a function of chromosomal location (A) and relative fitness as a function of relative *tufB* expression level (B).

The *tufB* operon is regulated under natural circumstances to react to changes in the cellular EF-Tu level (21). This regulation is most likely able to compensate for reduced *tufB* gene dosage in the strains with a translocated *tufB* operon (Table 1). To test this hypothesis, we deleted the *tufA* gene from each strain, thus constructing a set of strains whose only source of EF-Tu is from the *tufB* gene. Under these conditions, the constant cellular EF-Tu starvation conditions should lead to constitutive expression from the *tufB* operon at its maximum, and the gene dosage effect should be visible (due to the loss of the regulatory buffer). Measurements of the growth rate for each of the strains showed a significant reduction in fitness associated with displacement of the *tufB* operon in the three nonnative locations that were moved further away from the origin of replication (*P* < 0.0001) and a slight increase when moved closer toward the origin (*P* < 0.01) (Table 1). There is a positive correlation between the distance of the *tufB* operon from OriC and the magnitude of the decrease in fitness (*R*^2^ = 0.98) (Fig. 2), such that for each additional 100 kb from the origin of replication, the growth rate decreased by approximately 1.6%. The naive assumption was that the observed reduction in cellular fitness is the result of a reduced gene dosage for locations that are more distant from the origin of replication. To test this assumption, we measured EF-Tu expression levels based on a transcriptional *tufB*-*yfp* fusion assay (Materials and Methods) for each of the five chromosomal locations. The results are consistent with the gene dosage hypothesis, showing that EF-Tu expression levels are a function of distance to the origin of replication (Fig. 3A) and that reduced EF-Tu levels lead to a reduction in cellular fitness (Fig. 3B). The overall conclusion from these experiments is that chromosomal location does affect expression from the *tufB* operon (via gene dosage) but that the operon has the natural ability to adjust its expression levels to buffer gene location effects.

### Adjustment of expression level can overcome gene dosage effects.

The results thus far show that bacterial growth rate is reduced when the *tufB* operon is translocated to chromosomal locations that are more distant from the origin of replication but that the operon has the intrinsic ability to upregulate its expression to compensate and buffer the effects of reduced gene dosage. We next asked whether bacteria suffering the effects of reduced gene dosage, where the *tufB* operon is expressed at its regulatory maximum (equivalent to an operon without regulation), could evolve to reduce these fitness effects. To study this, we experimentally evolved a subset of the constructed strains with *tufB* displaced and *tufA* deleted, for a total of 500 generations each, by serial transfer in Luria-Bertani broth (LB). Seven independent lineages of each of the strains carrying the *tufB* operon at locations 1555 kb and 2154 kb distance to the origin of replication (chosen because they had the largest fitness costs associated with displacement) were experimentally evolved to enrich for mutants that had an improved growth rate. In addition, six independent lineages of an isogenic strain with the *tufB* operon at its native location (lacking *tufA*) were also evolved for 500 generations, as a control to identify mutations that might arise as a function of medium adaptation and/or to compensate for the lack of *tufA*. We isolated one evolved clone from each lineage for further analysis after 500 generations of evolution. With only one exception, all 14 evolved isolates showed a significantly improved relative fitness. The improvement in growth rate for the lineages with the *tufB* operon displaced to a nonnative location was up to 43%, and all lineages fully compensated for the fitness cost caused by the *tufB* operon translocation relative to the nonevolved parental strain (see Table S1 in the supplemental material). To understand how the evolved lineages had increased their growth rates, we analyzed the isolates from each evolved lineage by whole-genome sequencing. We included the evolved control strains with the *tufB* operon at its native location to identify mutations selected either for medium adaptation or as a compensation for the deletion of the *tufA* gene. These control strains had acquired mutations in *treB*, *flhC*, and *flhD*, genes that have been previously associated with adaptation to growth in LB or other laboratory media (22). Similar mutations were identified in each of the evolved strains with a displaced *tufB* operon, consistent with medium adaptation mutations also occurring in these lineages. In addition to these medium adaptation mutations, each of the evolved clones with the *tufB* operon at a nonnative location had acquired genetic changes affecting the *tufB* operon directly. These changes included amplifications of a chromosomal region that contained the *tufB* operon and/or the acquisition of single point mutations within the operon itself (Table 2). The common feature of the amplifications was the increase in copy number of the *tufB* operon, but otherwise, they were genetically diverse and had many different junction points (Tables S1 and S2). The data are consistent with selection of amplifications being driven by selection for an increased level of EF-Tu but do not exclude selection for increased levels of the associated tRNA genes (but see below). The point mutations identified in the *tufB* operon are all located close to or at the beginning of the *tufB* coding sequence in a region previously shown to be important for regulating the level of *tufB* expression (21). Indeed, all identified mutations had previously been shown to be mutations that increase expression of *tufB* leading to increased production of EF-Tu (21). No mutations were selected in any of the four tRNA genes or in the promoter or upstream regulatory region of the *tufB* operon. The locations of the point mutations, and the frequent occurrence of amplifications of the *tufB* operon, support the hypothesis that a large part of the fitness cost associated with *tufB* operon displacement is due to the production of insufficient EF-Tu to support the maximum growth rate. Thus, the primary focus of the selected genetic changes in the evolution experiment was on the acquisition of mutations that are predicted to increase the level of EF-Tu. Taken together, these results show the existence of a mutational buffer for genes that are affected by decreased gene dosage (due to translocation), as such mutations are able to upregulate expression to compensate for the reduced effective gene copy number.

**TABLE 2:** Genetic changes identified in the *tufB* operon of evolved strains

Genetic change	*N*	Relative expression^*a*^
Amplifications that include the *tufB* operon	6	ND
*tufB* C-60T	1	1.31
*tufB* G-29A	2	1.73
*tufB* G-29T	1	1.62
*tufB* G-29C	1	1.33
*tufB* C36T	4	1.48

a Expression level data from Brandis et al. (21). ND, not determined.

10.1128/mBio.01446-18.1 TABLE S1 Fitness data and genotypes of evolved isolates. Download Table S1, DOCX file, 0.02 MB. Copyright © 2018 Garmendia et al. 2018 Garmendia et al. This content is distributed under the terms of the Creative Commons Attribution 4.0 International license.

10.1128/mBio.01446-18.2 TABLE S2 Details of the amplifications found in the evolved clones. Download Table S2, DOCX file, 0.01 MB. Copyright © 2018 Garmendia et al. 2018 Garmendia et al. This content is distributed under the terms of the Creative Commons Attribution 4.0 International license.

## DISCUSSION

The organization of genes on the chromosome of *Salmonella* is consistent with the hypothesis that highly expressed genes that are important for fast growth are preferentially located in the OriC-proximal part of the chromosome where expression levels can be increased by taking advantage of the gene dosage effects of overlapping cycles of chromosomal replication (3–6). Previous studies on gene dosage effects have focused on testing whether changes in gene copy numbers due to overlapping replication cycles can have an effect on expression levels of genes. To test this, constitutively expressed genes (5, 7) or genes that were constituitively expressed after induction (6, 8) were moved across the chromosome, and transcription levels were determined. These studies came to the conclusion that gene dosage does affect transcript levels and that the observed changes corelated well to changes in gene copy number. While demonstrating that chromosomal location does affect effective gene copy numbers, these studies do not address the biological significance of gene dosage. The use of the *hisO1242* allele (5) or IPTG to induce the *lac* promoter (8) removes the natural regulation of these promoters. Under natural circumstances, feedback regulation in these experiments might have changed the expression levels of each gene copy within the cell, leading to no change in total cellular expression regardless of location. Since the expression of highly expressed genes is usually regulated (21, 23–25), it is not obvious that changes in gene dosage translate into actual changes in total cellular expression levels. To test whether gene dosage effects are also visible in naturally expressed operons, we displaced the highly expressed *tufB* operon to multiple nonnative locations on the chromosome. Our data showed only a small effect on bacterial growth rate when the operon was displaced from its native location to locations closer to the terminus of replication. This result is of particular interest, since the *tuf* genes are among the most highly expressed genes within the genome (9, 17), and selection to take advantage of gene dosage effects would, therefore, be expected to be strong. These results are not necessarily in conflict with previous studies because the *tuf* genes are known to be regulated (21) and changes in expression levels could buffer the effects of changes in gene dosage. To validate our experimental system and show that our results complement previous studies on gene dosage rather than oppose them, we adjusted the experimental conditions to mimic those in previous reports. To this end, we removed the *tufA* gene from the bacterial chromosome, thus leading to an extreme EF-Tu starvation condition. This change virtually removes the regulation of the *tufB* gene because the constant EF-Tu starvation leads to its constitutive maximum transcription (14, 21). As expected, gene dosage effects were visible for the *tufB* operon under these circumstances, and our data showed a good correlation between distance from OriC and growth rate (Table 1 and Fig. 2). Furthermore, we were able to show that the changes in growth rates correlated well with changes in the expression of the *tufB* gene (Fig. 3B). These data indicate that there might be two classes of genes: (i) genes that are constitutively expressed and where gene dosage plays a major role in affecting transcript levels and (ii) genes that are under regulation and where gene dosage plays a minor or no selective role. rRNA operons in *Salmonella*, present in seven separate copies on the chromosome in addition to being located in the OriC-proximal part of the chromosome, are likely members of the first class, where gene copy number is strongly selected (26). It is not clear though to what extent any other genes are affected by gene dosage. The *tuf* genes, present in two separate chromosomal copies, would be obvious candidates, but our data indicate that chromosomal location affects cellular EF-Tu levels only to a minor degree when both genes are present in the chromosome. Another group of genes that could be affected by chromosomal location are ribosomal proteins, which are highly expressed, important for growth, and exist in only one copy on the chromosome; thus, they could be more affected by gene dosage. However, the cellular concentration of each ribosomal protein is approximately sevenfold lower than that of EF-Tu, which means that the required level of expression of each *tuf* gene is at least threefold higher than that of each ribosomal protein gene. It is, therefore, not obvious that there would be a strong selection for chromosomal location for reasons of gene dosage if the required expression levels could be achieved by selecting appropriate transcriptional or translational expression levels. Nevertheless, the great majority of ribosomal protein genes are located in the OriC-proximal part of the chromosome.

An essential prerequisite for the model that chromosomal location is selected to maximize expression levels of highly expressed genes (via gene dosage) is that an increase in gene copy number is the only way to increase expression levels to the required cellular level. This might be true for the rRNA operons in the *Salmonella* chromosome (as discussed above), but it is not clear that it would be the case for any other genes, especially protein coding genes, which can be regulated at both transcriptional and translational levels. If a gene were to be moved to a new location associated with a decrease in gene dosage, it should be possible to adjust cellular expression levels by increasing either transcription or translation, thus rendering most gene dosage effects insignificant. We tested this hypothesis for our system and evolved strains with translocated *tufB* operons and deleted *tufA* genes (EF-Tu starvation conditions) for increased growth rate. Our results showed that the gene dosage effect was very quickly ameliorated by genetic changes that increased the expression level of *tufB*. This was achieved by amplification of the *tufB* operon region and by point mutations within the operon that have previously been shown to significantly increase *tufB* expression (Table 2 and Fig. 2) (21). These data support an evolutionary interplay between gene location and the regulation of gene expression level, driven by the demand for a level of product sufficient to confer the selected phenotype (in this case, fast growth in rich nutrient conditions). This implies that the location of a gene on the chromosome will influence the absolute expression level required to support the function of the gene product (to adjust for gene dosage effects) but that the requirements for a particular level of product will not drive the selection of chromosomal location.

The organization of highly expressed genes on the *Salmonella* chromosome is clearly nonrandom, which implies the existence of an underlying selective force. Our data suggest that a gene dosage-dependent increase in expression level *per se* is not a strong selective force determining precise chromosomal locations, since natural operons can quickly adjust expression levels to compensate for changes in demand. We are, however, not ruling out any role for gene dosage in selecting location. It is possible that it is beneficial for the cell to have lower levels of transcription and translation from several copies of a gene than to have higher levels of expression from a single copy, for example if the level of expression is associated with increased mutation rates, or errors in the product (19). In such cases, gene dosage could be a selected parameter to maintain an optimal expression level of some highly expressed genes.

**FIG 4:**
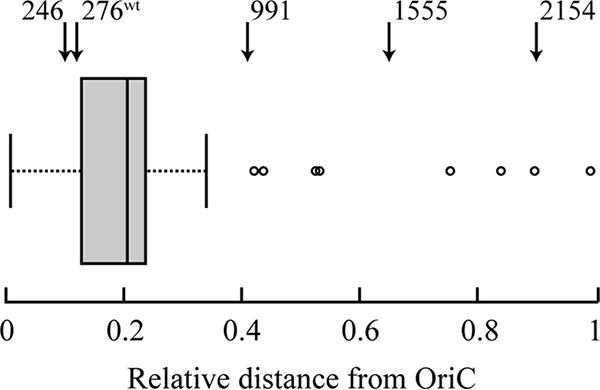
Distribution of highly expressed genes on the *Salmonella* chromosome. The arrows at the top of the figure indicate the locations of the wild-type (wt) and four displaced *tufB* operons.

An alternative theory is that gene location bias is selected for regulatory functions, rather than to support high expression levels. An analysis of the location of genes that are part of the transcription and translation machinery (rRNAs, ribosomal proteins, elongation factors, RNA polymerase subunits) shows that the majority of these genes are located in a small region around 20% of the linear distance between the origin and terminus of replication (Fig. 4). In addition to this linear relationship, conformation capture studies have suggested a spatial assembly in transcription foci of highly expressed genes in exponential phase (27). One particular example of this is the colocalization of six out of seven *rrn* operons in the E. coli chromosome, as seen by fluorescence microscopy (28).

This bias in location ensures that the genes that are important for transcription and translation are synchronized in the same replication state, and expression levels (via gene dosage effects) are directly linked to the growth speed of the bacteria. Gene dosage effects could, therefore, be seen as a regulatory mechanism that simultaneously links the expression levels of a multitude of essential genes to the bacterial growth state. In this case, moving the location of a single operon would not be expected to result in a significant selective disadvantage.

## MATERIALS AND METHODS

### Media and growth conditions.

Luria-Bertani broth (LB) containing per liter, 10 g NaCl (5 g in the case of low-salt LB), 5 g yeast extract, and 10 g tryptone, was used as liquid medium for growing bacterial cultures. LA (LB broth with 1.5% agar) was used as the solid medium. For *sacB* counterselections, LB and LA contained no NaCl, and the LA medium was supplemented with sucrose to 5%. All incubations were made at 37°C (unless stated otherwise), and liquid cultures were shaken at 200 rpm for aeration. Antibiotics were purchased from Sigma-Aldrich (Sweden) and used at the following final concentrations: kanamycin, 25 mg liter^−1^; chloramphenicol, 30 mg liter^−1^; tetracycline, 15 mg liter^−1^.

### Bacterial strains.

All the strains used in this work are derivatives of the sequenced Salmonella enterica serovar Typhimurium, strain LT2 (26), referred to in the text as *S. Typhimurium*. A complete list of strain genotypes is shown in Table S3 in the supplemental material.

10.1128/mBio.01446-18.3 TABLE S3 Strain list. Download Table S3, DOCX file, 0.01 MB. Copyright © 2018 Garmendia et al. 2018 Garmendia et al. This content is distributed under the terms of the Creative Commons Attribution 4.0 International license.

### Strain construction.

All engineered insertions into the chromosome in this study were made by double-stranded DNA lambda-red recombineering (29, 30). The lambda-red genes were induced in each strain from the temperature-sensitive pSIM5-tet (tet stands for tetracycline) plasmid (31) by incubation of an over-day culture (optical density at 600 nm [OD_600_] of 0.3) at 43°C for 15 min. After cooling for 10 min on ice, the cells were made electrocompetent by washing them in ice-cold water three times. The electroporation of the double-stranded DNA was done using a Gene Pulser (Bio-Rad, USA) by mixing 50 µl of electrocompetent cells and 100 ng of DNA, with the settings 1.8 kV, 25 µF, and 200 Ω. Cells were recovered in 1 ml of low-salt LB at 30°C overnight with aeration, and after recovery, 100 µl of culture was spread onto LA plates containing the appropriate antibiotic for selection of recombinants.

Genetic markers were moved between strains by transduction using the bacteriophage P22 HT105 *int*^−^ (32). A P22 lysate grown on donor strains was made by mixing 1 ml of bacterial culture (OD_600_ ≈ 0.6) with 100 µl of P22 lysate grown on wild-type LT2. Four milliliters of soft agar (LB medium plus 0.8% agar) was added to the mixture and then poured onto LA plates. After an overnight incubation, the soft agar was scraped off the plate, mixed with 4.5 ml of LB, and vortexed until a slurry was obtained. The tubes were centrifuged for 15 min (3,000 *× g*), and the supernatant was filtered through a 0.2-µm filter to obtain a P22 lysate. A lysate was used to transduce recipient strains by mixing 1 ml of overnight culture of the recipient with 50 µl of the lysate and incubating the mixture for 1 h. One hundred microliters of each mixture was then spread onto selective plates, which were incubated overnight at the appropriate temperature. Transductant colonies were picked from selective plates and purified from residual phage by restreaking for single colonies twice in succession on LA plates.

The *cat* gene was removed from the recombineered *tufB* operon in each strain by introduction of the pCP20 plasmid expressing Flp recombinase (33), leaving one FLP recombination target (FRT) scar at the site. The *kan-sacB-t0* and *cat-sacB* cassettes were removed from the chromosome by single-stranded lambda-red recombineering with counterselection for sucrose resistance (34), leaving no scar, following the same steps as stated above for the double-stranded DNA lambda-red recombineering.

### PCR and oligonucleotides.

PCR was performed using an S1000 thermal cycler (Bio-Rad, USA). Oligonucleotides were designed using the software CLC Main Workbench 7 (CLC bio, Denmark) with the *Salmonella* Typhimurium LT2 genome as the reference. For amplification of the long *cat-sacB* or *kan-sacB* cassette, PCRs were performed using Phusion high-fidelity PCR master mix with HF buffer (New England Biolabs, USA) under the following cycling conditions: (i) 30 s at 98°C; (ii) 30 cycles, with 1 cycle consisting of 10 s at 98°C, 30 s at 55°C, and 3 min at 72°C; (iii) 7 min at 72°C. For routine PCR amplifications, Fermentas PCR master mix (Thermo Scientific, USA) was used under the following cycling conditions: (i) 5 s at 95°C; (ii) 30 cycles, with 1 cycle consisting of 30 s at 95°C, 30 s at the annealing temperature (T_A_), and 72°C for elongation time (E_T_); (iii) 5 min at 72°C. T_A_ varied depending on the pair of primers used, and E_T_ was based on the length of the expected product (30 min per kb). A list of the oligonucleotide sequences is available in Table S4.

10.1128/mBio.01446-18.4 TABLE S4 Sequences of oligonucleotides used for construction of strains by recombineering, for PCR, and as primers for DNA sequencing. Download Table S4, DOCX file, 0.11 MB. Copyright © 2018 Garmendia et al. 2018 Garmendia et al. This content is distributed under the terms of the Creative Commons Attribution 4.0 International license.

### Local DNA sequencing.

Local sequencing of PCR-amplified products was performed at the Macrogen Europe sequencing facility (Amsterdam, The Netherlands), and data were analyzed using the software CLC Main Workbench 7 (CLC bio, Denmark).

### Experimental evolution.

Seven independent lineages of each of the strains were grown in 10-ml Falcon tubes in LB at 37°C overnight with aeration (200 rpm). All lineages were serially passaged after 24 h by transferring 2-μl portions of the overnight cultures into 2 ml of fresh LB medium, resulting in 10 generations of growth per passage. The relative fitness of each lineage was monitored every 100 generations by measuring growth rate using a Bioscreen C. All lineages were passaged for 500 generations and plated to obtain single colonies. One clone from each lineage was picked to measure fitness and for whole-genome sequence analysis.

### Whole-genome sequencing and analysis.

Genomic DNA was prepared using the MasterPure DNA purification kit (Epicentre Biotechnologies, USA). Final prepared DNA was diluted in EB buffer, and concentrations were measured using a Qubit 2.0 fluorometer (Invitrogen via ThermoFisher Scientific). To create libraries of paired-end fragments, Nextera XT sample preparation kit (Illumina, USA) was used following instructions from the manufacturer. Sequencing was performed on an Illumina MiSeq instrument, generating 250-bp paired-end reads. Whole-genome sequencing data were analyzed using the software CLC Genomic Workbench (CLC bio, Qiagen, Denmark).

### Doubling time and relative fitness measurements.

Doubling times of bacterial cultures were measured by monitoring the rate of increase in optical density using a Bioscreen C machine (Oy Growth Curves Ab Ltd., Finland), growing the cultures in a Honeycomb microtiter plate. To set up an experiment, 0.5 μl of overnight culture from a single colony was diluted into 1 ml of LB medium. For each independent colony, 3 wells of the microtiter plate were filled with 280 μl of each dilution, as technical replicates. Cultures were grown for 18 h at 37°C with continuous shaking, and readings of OD_600_ were taken at 5-min intervals. The doubling time (*DT*) of each strain during exponential growth was estimated over an interval of 50 min in the linear region of the curve, using the equation *DT* = ln 2/slope. The relative fitness of each strain was calculated by comparing the independent *DT* measurements to the average of the measurements of the reference strain.

### *tufB* expression analysis.

Expression of *tufB* was measured using transcriptional *tufB-yfp* fusions. Overnight cultures were grown in 2 ml LB and incubated at 37°C with continuous shaking. One microliter of culture was transferred into 200 µl of phosphate-buffered saline (PBS), and the average yellow fluorescent protein (YFP) fluorescence of 10^5^ cells was measured using flow cytometry (MACSQuant VYB). All values are shown as averages ± standard deviations of five independent measurements relative to the *tufB* expression at the native chromosomal location.

### Statistical analysis.

All statistical analyses were performed using GraphPad Prism v6.0c (GraphPad Software, USA). The significance of differences between growth rates was calculated using an unpaired two-tailed *t* test.
