# Supplementary material for: Transcriptional Regulation Buffers Gene Dosage Effects on a Highly Expressed Operon in Salmonella
Source: mBio. 2018 Sep 11;9(5):e01446-18. doi: 10.1128/mBio.01446-18 (PMC6134099; doi:10.1128/mBio.01446-18)
Supplement: TABLE S1 [file mbo004184058st1.docx]

**TABLE S1** Fitness data and genotypes of evolved isolates

| **Isolate ^a^** | **Fitness ^b^** | **Genotype ^c^** |
| --- | --- | --- |
| 276 ^d^ | 1.00 ± 0.02 | ∆*tufA* |
| 276-1 | 1.16 ± 0.03 | as 276, *flhC* Gly55Trp |
| 276-2 | 1.19 ± 0.03 | as 276, *flhC* Arg37Cys |
| 276-3 | 1.15 ± 0.02 | as 276, *treB* nt534 insT |
| 276-4 | 1.17 ± 0.02 | as 276, *flhC* Leu83fs |
| 276-5 | 1.25 ± 0.06 | as 276, ***tufB* C36T**, *flhD* Met4Thr |
| 276-6 | 1.16 ± 0.03 | as 276, *flhC* Arg119Cys |
|  |  |  |
| 1555 ^d^ | 0.80 ± 0.01 | ∆*tufA*, ∆*thrU*-*tyrU*-*glyT*-*thrT*-*tufB*, *STM0715*<>*tufB* operon |
| 1555-1 | 1.12 ± 0.03 | as 1555, ***tufB* G-29C**, ∆42bp between *STM2584*/*5* |
| 1555-2 | 1.12 ± 0.03 | as 1555, ***tufB* C-60T** |
| 1555-3 | 1.17 ± 0.02 | as 1555, **35 kb amplification**, *flhC* Trp121insArgAla |
| 1555-4 | 1.20 ± 0.04 | as 1555, ***tufB* G-29A**, *flhD* Tyr20Asp |
| 1555-5 | 1.15 ± 0.02 | as 1555, ***tufB* G-29T**, *treB* nt534 insT |
| 1555-6 | 0.79 ± 0.03 | as 1555, **927 kb amplification**, *ftsW* Ser388Pro, *crl* Phe35fs |
| 1555-7 | 1.13 ± 0.02 | as 1555, ***tufB* C36T**, 12 kb deletion ^e^, *poxB* G-77C |
|  |  |  |
| 2154 ^d^ | 0.73 ± 0.02 | ∆*tufA*, ∆*thrU*-*tyrU*-*glyT*-*thrT*-*tufB*, *STM1300*<>*tufB* operon |
| 2154-1 | 1.16 ± 0.02 | as 2154, **61 kb amplification**, *treB* nt534 insT |
| 2154-2 | 1.08 ± 0.05 | as 2154, **107 kb amplification**, *tolC* Lys152Glu |
| 2154-3 | 1.11 ± 0.02 | as 2154, 68 **kb amplification** |
| 2154-4 | 1.12 ± 0.02 | as 2154, *ftsL* Leu24fs, *fimY* A-86G |
| 2154-5 | 1.09 ± 0.02 | as 2154, 8 kb deletion ^f^ |
| 2154-6 | 1.14 ± 0.03 | as 2154, **112 kb amplification** |
| 2154-7 | 1.04 ± 0.03 | as 2154, *treB* nt534 insT, *sfsA* *235Gln, *phrB* Ala65Ser, *rpoS* Arg322cys |

^a^ Chromosomal location as kb distance to OriC, location 276 is the native *tufB* operon location.

^b^ Fitness ± standard deviation relative to the un-evolved isogenic wild type (∆*tufA*) measured as exponential growth rate.

^c^ Changes that directly involve *tufB* are shown in bold. Details of amplifications are shown in Table S2.

^d^ Un-evolved parental strains.

^e^ Inactivation of *cheAWMRB*, *motAB*, *flhDC*, *yecG*, *otsAB*.

^f^ Inactivation of *uvrYC*, *yecF*, *sdiA*, *yecSC*, *yedO*, *fliAZY*.
