# Supplementary material for: Transcriptional Regulation Buffers Gene Dosage Effects on a Highly Expressed Operon in Salmonella
Source: mBio. 2018 Sep 11;9(5):e01446-18. doi: 10.1128/mBio.01446-18 (PMC6134099; doi:10.1128/mBio.01446-18)
Supplement: TABLE S2 [file mbo004184058st2.docx]

**TABLE S2** Details of the amplifications found in the evolved clones

| **Isolate** | **Length** | **Amplification level** | **Junctions points ^a^** |
| --- | --- | --- | --- |
| 1555-3 | 35 kb | 4x | 776307-811339 |
| 1555-6 | 927 kb | 2x | 542461-1469786 |
| 2154-1 | 61 kb | 4x | 1368646-1429537 |
| 2154-2 | 107 kb | 3x | 1327990-1434953 |
| 2154-3 | 68 kb | 3x | 1358629-1427122 |
| 2154-6 | 112 kb | 3-5x | 1345742-1457914 |

^a^ Numbering in reference to *Salmonella* Typhimurium LT2 genome NC_003197
