# Supplementary material for: Transcriptional Regulation Buffers Gene Dosage Effects on a Highly Expressed Operon in Salmonella
Source: mBio. 2018 Sep 11;9(5):e01446-18. doi: 10.1128/mBio.01446-18 (PMC6134099; doi:10.1128/mBio.01446-18)
Supplement: TABLE S3 [file mbo004184058st3.docx]

**TABLE S3** Strain list

| **Strain** | **Relevant Genotype** |
| --- | --- |
| TH4527 | *S. typhimurium* LT2 |
| TH9977 | *∆tufA* |
| TH9978 | *∆tufB* |
| TH9881 | Δ*thrU*-*tyrU*-*glyT*-*thrT*-*tufB*, *STM4002*<>*tufA*_ter-FRT-*tufB*_operon |
| TH9335 | Δ*thrU*-*tyrU*-*glyT*-*thrT*-*tufB*, *sfsA*<>*tufA*_ter-FRT-*tufB*_operon |
| TH9341 | Δ*thrU*-*tyrU*-*glyT*-*thrT*-*tufB*, *STM0715*<>*tufA*_ter-FRT-*tufB*_operon |
| TH9337 | Δ*thrU*-*tyrU*-*glyT*-*thrT*-*tufB*, *STM1300*<>*tufA*_ter-FRT-*tufB*_operon |
| TH9951 | Δ*tufA*, Δ*thrU*-*tyrU*-*glyT*-*thrT*-*tufB*, *STM4002*<>*tufA*_ter-FRT-*tufB*_operon |
| TH9430 | Δ*tufA*, Δ*thrU*-*tyrU*-*glyT*-*thrT*-*tufB*, *sfsA*<>*tufA*_ter-FRT-*tufB*_operon |
| TH9436 | Δ*tufA*, Δ*thrU*-*tyrU*-*glyT*-*thrT*-*tufB*, *STM0715*<>*tufA*_ter-FRT-*tufB*_operon |
| TH9432 | Δ*tufA*, Δ*thrU*-*tyrU*-*glyT*-*thrT*-*tufB*, *STM1300*<>*tufA*_ter-FRT-*tufB*_operon |
| TH11209 | *tufB*_operon-*yfp* |
| TH11213 | Δ*thrU*-*tyrU*-*glyT*-*thrT*-*tufB*, *STM4002*<>*tufA*_ter-FRT-*tufB*_operon-*yfp* |
| TH11210 | Δ*thrU*-*tyrU*-*glyT*-*thrT*-*tufB*, *sfsA*<>*tufA*_ter-FRT-*tufB*_operon-*yfp* |
| TH11212 | Δ*thrU*-*tyrU*-*glyT*-*thrT*-*tufB*, *STM0715*<>*tufA*_ter-FRT-*tufB*_operon-*yfp* |
| TH11211 | Δ*thrU*-*tyrU*-*glyT*-*thrT*-*tufB*, *STM1300*<>*tufA*_ter-FRT-*tufB*_operon-*yfp* |
| TH11105 | Δ*tufA*, *tufB*_operon-*yfp* |
| TH11114 | Δ*tufA*, Δ*thrU*-*tyrU*-*glyT*-*thrT*-*tufB*, *STM4002*<>*tufA*_ter-FRT-*tufB*_operon-*yfp* |
| TH11106 | Δ*tufA*, Δ*thrU*-*tyrU*-*glyT*-*thrT*-*tufB*, *sfsA*<>*tufA*_ter-FRT-*tufB*_operon-*yfp* |
| TH11108 | Δ*tufA*, Δ*thrU*-*tyrU*-*glyT*-*thrT*-*tufB*, *STM0715*<>*tufA*_ter-FRT-*tufB*_operon-*yfp* |
| TH11110 | Δ*tufA*, Δ*thrU*-*tyrU*-*glyT*-*thrT*-*tufB*, *STM1300*<>*tufA*_ter-FRT-*tufB*_operon-*yfp* |
