# Supplementary material for: Transcriptional Regulation Buffers Gene Dosage Effects on a Highly Expressed Operon in Salmonella
Source: mBio. 2018 Sep 11;9(5):e01446-18. doi: 10.1128/mBio.01446-18 (PMC6134099; doi:10.1128/mBio.01446-18)
Supplement: TABLE S4 [file mbo004184058st4.docx]

**TABLE S4** Sequences of oligonucleotides used for construction of strains by recombineering, for PCR and as primers for DNA sequencing

| Name | Sequence (3'-5') |
| --- | --- |
| *Amplify FRT-Cat-FRT and insert tufA terminator-FRT-Cat-FRT in front of tufB operon* | |
| cmR_tufBop_fw | CCCTGAGAAGCAATGTGACGAATATACCAGATACAGAAGAGGGCGCTTCGGCGCCCTTTTTTGAAGTTCCTATACTTTCTAGAGAATAGGAACTTCCACCAAACACCCCCCAAAACC |
| cmR_tufBop_rv | ATACGACGCGGCATTTCAACGCTCCGTCGATAGAAAGCAAGAAGTTCCTATTCTCTAGAAAGTATAGGAACTTCACACACAACCACACCACACCAC |
|  |  |
| *Amplify custom-made tufB operon and insert it in the selected positions and orientations* | |
| sfsA<>tufBop_fw | GTCCGGGCGGAAATCTGCCTGTAACATAAAATCTATACGGGGGCGCTTCGGCGCCCTTTTTT |
| sfsA<>tufBop_rv | ATATTCTGAAAAGTGAAGTCAAATATGGCGCCGAGCGCAGGTTCTGGTGTGAAAGCGTGC |
| STM1300<>tufbop_fw | GATATTCAGGTGAATGTCCCGCCGGAAGTTTTCAGCACGCGGGCGCTTCGGCGCCCTTTTT |
| STM1300<>tufbop_rv | TAAACGCAGCACTGAATGCAGGGCTGGGACGACTGACCGCGTTCTGGTGTGAAAGCGTGC |
| STM4002<>tufBop_fw | GGTCAGGAGCGCCAGGAGGCGAAGACACAGGATTGTCAGGGGGCGCTTCGGCGCCCTTTTT |
| STM4002<>tufBop_rv | TTTCCATTTCCTTTTACTAACGTCTCCGGACGTTTGTCTTGTTCTGGTGTGAAAGCGTGC |
| STM0715<>tufBop_fw | CCAGATTGATTCGCTGTTTCACTATCACGTCATGGATAGCGGGGCGCTTCGGCGCCCTTT |
| STM0715<>tufBop_rv | GCGTAAACAGCTTAAAATCCATATAGCTGGCAATCTGGCAGTTCTGGTGTGAAAGCGTGC |
|  |  |
| *Amplify and kan-sacB-t0 and replace native tRNAs from tufB operon* | |
| tRNAs_kan_sacB_fw | GGGGAGGAATAATAAGAAAAAATCTCGCTGTGAAAGCTATAAAATGAGACGTTGATCGGCACG |
| tRNAs_kan_sacB_rv | ATGCCCTTTTATTGCGCATTGCGTCAAATGTTATCAGCGAATCAAAGGGAAAACTGTCCA |
|  |  |
| *Scar-free deletion of native tufB operon* | |
| tRNAs_repair | CTTTTATTGCGCATTGCGTCAAATGTTATCAGCGAATAGCTTTCACAGCGAGATTTTTTCTTATTATTCC |
|  |  |
|  |  |
| *Scar-free deletion of cat-sacB in tufA* | |
| del_catsacB_tufA | GCGCCCTCTTCAATTCAAAACTAATTAACGAGTAAGGCTATATTCCTTACTATAGCGCTCTCCCCTTCAG |
|  |  |
| *Check presence/absence of the specific changes made in the chromosome* | |
| sfsA_fw | TATGGGTACTGGTTCGTT |
| sfsA_rv | GCTATTTTCACCCCCTTT |
| 1300_fw | AGCGATGACAAAAGAGGA |
| 1300_fw | TGGCCGACGAATTCATTT |
| 4002_fw | CATACGCTTTGTGAGACATT |
| 4002_rv | TCTGCCGCCTTTTTTCTT |
| 0715_fw | ACCTGACTCTTTATTACCCCT |
| 0715_rv | CAATATCCGACAAGCGCA |
| tRNAs_fw | TGTAGGTAAGGCGTCATT |
| tRNAs_rv | TCGCGATAGAGGTAGTTG |
| UL | CACCAAACACCCCCCAAAACC |
| UR | CACACAACCACACCACACCAC |
| kan_out | GTCATAGCCGAATAGCCTCTCCAC |
| cat_out | GGCGGGCAAGAATGTGAATAAAGG |
| sacB_out | GCTGTACCTCAAGCGAAAGG |
